# Complete Genome Sequence of *Salmonella enterica* subsp. *enterica* Serovar Paratyphi B Sequence Type 28 Harboring *mcr-1*

**DOI:** 10.1128/genomeA.00991-17

**Published:** 2017-09-14

**Authors:** Maria Borowiak, Jens A. Hammerl, Jennie Fischer, Istvan Szabo, Burkhard Malorny

**Affiliations:** German Federal Institute for Risk Assessment (BfR), Department for Biological Safety, Berlin, Germany

## Abstract

In 2015, plasmid-mediated colistin resistance was reported to be caused by a mobilized phosphoethanolamine transferase gene (*mcr-1*) in *Enterobacteriaceae*. Here, we announce the complete genome sequence of the earliest *d*-tartrate-fermenting *Salmonella enterica* subsp. *enterica* serovar Paratyphi B isolate harboring *mcr-1* from the collection of the German National Reference Laboratory for Salmonella.

## GENOME ANNOUNCEMENT

A common cause of gastroenteritis worldwide is *d*-tartrate-fermenting (*d*Ta+) *Salmonella enterica* subsp. *enterica* serovar Paratyphi B (formerly known as *S. enterica* serovar Java) (1). Since the late 1990s, a distinct multidrug-resistant genetic lineage of this zoonotic serovar has been commonly isolated from poultry and poultry products, especially in Germany, the Netherlands, and Belgium (2). Multidrug resistance to up to six classes of antimicrobials of the lineage is caused by a chromosomally located class 2 integron carrying the *dfrA1-sat2-aadA1* (Tn*7*) array of gene cassettes (conferring resistance to trimethoprim, streptothricin, and aminoglycosides), a mutation in the quinolone resistance-determining region (QRDR) of the *gyrA* gene (conferring resistance to quinolones), and occasional acquisition of additional resistance plasmids, including extended-spectrum β-lactamase (ESBL) genes (3, 4). In 2015, the first mobilized colistin resistance gene (*mcr-1*) was identified in *Escherichia coli* isolates from China (5) and later reported in a number of other countries worldwide (6). These findings raised a significant public health concern because colistin belongs to the last-resort antibiotics used to treat multidrug-resistant infections. 

PCR screening of isolates from the collection of the German National Reference Laboratory for Salmonella revealed that *S*. Paratyphi B (*d*Ta+) acquired the *mcr-1* gene in the year 2008. Our earliest observed *mcr-1*-positive isolate (08-00436) originated from chicken skin. 

A single colony of isolate 08-00436 was picked and cultured for 16 h at 37°C in lysogeny broth supplemented with 2 mg/liter colistin-sulfate (Sigma-Aldrich, Darmstadt, Germany). Genomic DNA was extracted using the PureLink genomic DNA minikit (Invitrogen, Carlsbad, CA, USA). Genome sequencing was performed by the GATC Biotech AG (Constance, Germany) using the PacBio RSII system. A *de novo* genome assembly based on 55,146 reads from a single-molecule real-time (SMRT) cell (*N*_50_ read length, 21,347 bp; mean read length, 14,478 bp; mean read score, 0.86) was performed using the SMRT Analysis (version 2.3.0) software (Pacific Biosciences, USA), resulting in 3 contigs with an average coverage of 143.12× per consensus base. Short-read sequencing using Illumina MiSeq technology was carried out on the same DNA sample. Paired reads were mapped against PacBio contigs using CLC Genomics Workbench 9.5.2. The contigs were closed to circular molecules, and errors in homopolymeric regions were corrected. The resulting genome contains a chromosome (4,751,926 bp) and two plasmids, pSE08-00436-1 (264,914 bp) and pSE08-00436-2, (54,067 bp) comprising an average G+C content of 52%. 

Genome analysis using tools of the Center for Genomic Epidemiology (http://www.genomicepidemiology.org) revealed that the isolate belonged to the multilocus sequence type (MLST) 28 (ST28) and carries two copies of the class 2 integron, as well as additional resistance genes in the chromosome. Further resistance genes, including *mcr-1*, were located on the multireplicon IncHI2/IncHI2A/IncQ1 plasmid pSE08-00436-1. Initial genome annotation was performed using the automated Prokaryotic Genome Annotation Pipeline (https://www.ncbi.nlm.nih.gov/genome/annotation_prok/). Analysis of the results revealed 5,071 coding sequences (CDSs) (4,898 coding genes and 173 pseudogenes), as well as 117 RNA genes (22 rRNAs, 83 tRNAs, and 12 noncoding RNAs). Further assessment of the genes involved in resistance, plasmid transfer, and virulence is in progress.

### Accession number(s).

Sequences were deposited in GenBank under the accession numbers CP020492 (chromosome), CP020493 (pSE08-00436-1), and CP020494 (pSE08-00436-2).
